# DNA Damage Triggers Genetic Exchange in *Helicobacter pylori*

**DOI:** 10.1371/journal.ppat.1001026

**Published:** 2010-07-29

**Authors:** Marion S. Dorer, Jutta Fero, Nina R. Salama

**Affiliations:** Division of Human Biology, Fred Hutchinson Cancer Research Center, Seattle, Washington, United States of America; University of Illinois, United States of America

## Abstract

Many organisms respond to DNA damage by inducing expression of DNA repair genes. We find that the human stomach pathogen *Helicobacter pylori* instead induces transcription and translation of natural competence genes, thus increasing transformation frequency. Transcription of a lysozyme-like protein that promotes DNA donation from intact cells is also induced. Exogenous DNA modulates the DNA damage response, as both *recA* and the ability to take up DNA are required for full induction of the response. This feedback loop is active during stomach colonization, indicating a role in the pathogenesis of the bacterium. As patients can be infected with multiple genetically distinct clones of *H. pylori*, DNA damage induced genetic exchange may facilitate spread of antibiotic resistance and selection of fitter variants through re-assortment of preexisting alleles in this important human pathogen.

## Author Summary

All organisms have genetic programs to respond to stressful conditions. The human stomach pathogen, *Helicobacter pylori*, survives on the surface of the stomach lining for the lifetime of its host and causes a chronic inflammatory response. In this niche, *H. pylori* is likely exposed to constant DNA damage and requires DNA repair systems to survive in the host. Many bacteria encode a genetic program for a coordinated response to DNA damage called the SOS response, which typically includes transcriptional induction of DNA repair systems and mutagenic DNA polymerases and a temporary halt to cell division. This study demonstrates that *H. pylori* has a distinct DNA damage response: instead of activating DNA repair systems, it induces both DNA uptake machinery and an enzyme that liberates DNA from neighboring cells. This capacity for genetic exchange enhances recombination of exogenous DNA into the genome, thus contributing to both the high genetic diversity observed between *H. pylori* clinical isolates and the spread of antibiotic resistance.

## Introduction

The Gram-negative human stomach pathogen *Helicobacter pylori* occupies an exposed niche on the surface of the stomach epithelium and faces a chronic inflammatory response. Despite these challenges, *H. pylori* colonizes 50% of the world's population and chronic infection leads to gastritis, peptic ulcer disease, gastric cancer and gastric mucosal-associated lymphoid tissue lymphoma in a subset of infected patients [1]. Eradication requires a 7–14 day course of multiple antibiotics [2] and treatment failure due to chromosomally encoded antibiotic resistance is common [3].

Comparative genomic studies of *H. pylori* strains from diverse global populations revealed extensive genetic diversity in nucleotide sequence and gene content as well as a genome in linkage equilibrium [4], [5], [6], [7]. Allelic diversity is generated by mutation. Some isolates of *H. pylori*, including the one used in this study, have a mutation rate similar to *E. coli* [8], [9], whereas other studies have found a 10–700 fold higher mutation frequency in clinical isolates that may drive strain variation [10], [11]. Although infection of humans with a single strain is most common, mixed infection with distinct strains and genetic exchange between strains is observed [12], [13], [14], [15], [16], [17]. Most clinical isolates show natural competence for DNA uptake and recombination into the genome [18] which likely contributes to the recombination signatures observed in *H. pylori* genomes [4]. Exogenous DNA is transported into the cell by the Com type IV secretion system (T4SS), which is homologous to type IV systems found in *Agrobacterium tumefaciens* and other Gram-negative species [19]. These systems are known to transport DNA and proteins, but *H. pylori* is the only species known to use a T4SS for natural competence [20]. Unlike other organisms which carefully regulate competence, *H. pylori* competence is constitutive during logarithmic growth [21]. Thus, new alleles created by mutation can spread and re-assort by DNA exchange during mixed infection generating the heterogeneous populations observed in clinical isolates [4].

All organisms encode genetic programs for response to stressful conditions including DNA damage. In *H. pylori*, homologous recombination is required for resistance to antimicrobial agents that create DNA double strand breaks (DSBs) such as ciprofloxacin and colonization of the mouse stomach [22], [23]. The AddAB helicase-nuclease complex resects DSBs and loads RecA onto single strand DNA (ssDNA), which then mediates strand exchange, leading to homologous recombination and repair [23]. The requirement of RecA plus AddAB for efficient stomach colonization suggests that in the stomach *H. pylori* is either exposed to DNA damage that must be repaired or requires some other recombination-mediated event.

In some bacterial species, DNA damage induces a transcriptional program called the SOS response, which can include genes involved in DNA repair, cell cycle control and low-fidelity polymerases. The SOS response is triggered when RecA binds ssDNA, thus activating its co-protease activity towards LexA, a transcriptional repressor [24]. Expression of many other genes is changed by DNA damage in a LexA-independent manner and the genes expressed vary by species [25], [26]. Genome sequencing revealed that *H. pylori* lacks *lexA*, low-fidelity polymerases, and an obvious cell cycle repressor, suggesting that *H. pylori* lacks the SOS response [5], [27]. Various Gram-negative and Gram-positive organisms also lack *lexA*, including *Campylobacter jejuni* and *Streptococcus pneumoniae*, but only limited studies of their responses to DNA damage are available. In response to a short pulse of DNA damage, *C. jejuni* induces several genes including *mfd*, which encodes a transcription-coupling repair factor [28]. In *S. pneumoniae*, DNA damage and other stresses induce genetic competence [29]. The conservation of LexA-independent responses is unclear and the transcriptional response to DNA damage in *H. pylori* has not been described. Identification of the complete set of DNA damage responsive genes thus promised to provide insight into how this important pathogen responds to DNA damage and adapts to its environment. This work indicates that induction of competence is a major component of the *H. pylori* response to DNA damage and suggests a close relationship between DNA damage and genetic diversification during stomach colonization.

## Results/Discussion

### The *H. pylori* transcriptional response to DNA damage is distinct from SOS

To define critical pathways for an *H. pylori* DNA damage response, cDNA based microarrays [7] were used to measure transcriptional changes in cells undergoing DNA damage. Wild-type cells were exposed to the antibiotic ciprofloxacin, which binds DNA gyrase, causing DSBs [30], and compared to untreated wild-type cells. Using Significance Analysis of Microarrays (SAM, [31]), we observed significant induction of 127 genes and repression of 170 genes in ciprofloxacin-treated cells relative to untreated cells (1% false discovery rate (FDR)) (Table S1).

To further define the response to DNA damage, transcriptional changes were similarly measured in cells lacking *addA*, which is required for DSB repair by homologous recombination [22]. It is likely that the Δ*addA* mutant accumulates unrepaired DNA damage, because cells lacking *addA* replicate 1.1 fold slower than wild- type cells, which translates to a 100-fold decrease in CFU after 20 generations (Figure S1A and [32]). In contrast, cells lacking DNA single strand break repair due to mutation of *recR* [32] replicated with the same efficiency as wild-type cells (Figure S1B). These data suggest that DSBs occur during growth in broth culture, and that therefore cells lacking DSB repair, including the Δ*addA* mutant, undergo chronic DNA damage. Microarray analysis revealed that the Δ*addA* mutant showed induction of 67 genes and repression of 167 genes, compared to wild-type cells during logarithmic growth using a 5% FDR (SAM) (Table S1). The Δ*addA* mutant showed weaker transcriptional induction than ciprofloxacin, necessitating use of a higher FDR, possibly because ciprofloxacin treatment causes acute DNA damage whereas the Δ*addA* mutant undergoes chronic damage. We also queried transcriptional changes in cells lacking single strand break repair (Δ*recR* mutant), but observed no significant changes in gene expression (Table S2). These observations suggest that lack of DSB repair in the Δ*addA* mutant causes transcriptional changes.

**Figure 1:**
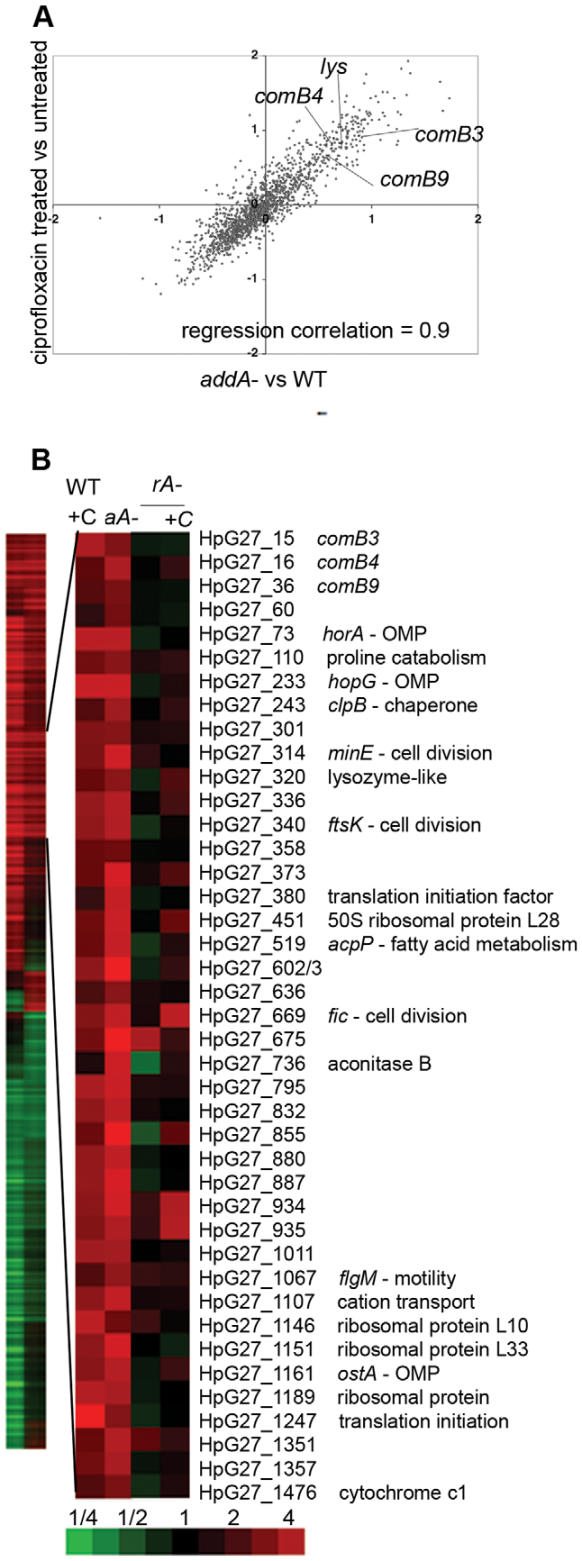
DNA damage induces natural competence. **A.** Ciprofloxacin exposure and *addA* mutation lead to similar transcriptional changes. A comparison of log_2_ ratios of gene expression changes measured by microarray for ciprofloxacin treatment (2.5 hours, 10× MIC) vs untreated and untreated Δ*addA* mutant vs wild-type cells during logarithmic growth. Each condition is represented as mean values measured from multiple microarray experiments from three independent cultures. Regression correlation = 0.9. **B.** RecA is required for induction of DNA damage responsive genes. Heat map showing 471 genes with 1.6 fold change upon treatment with ciprofloxacin (column 1) or in the Δ*addA* mutant (column 2). The enlarged panel shows genes with statistically significant induction in both ciprofloxacin-treated cells and the Δ*addA* mutant, as determined by SAM (DNA damage responsive genes), with annotation from strain G27 [46]. Untreated wild-type cells compared to either wild-type cells treated with ciprofloxacin (WT+C), untreated *ΔaddA* mutant (*aA−*), untreated Δ*recA* mutant (*rA−*) or the Δ*recA* mutant treated with ciprofloxacin (+C). Bottom, scale bar for heat maps in fold change. OMP = outer membrane protein, genes with no annotation are left blank.

Comparison of the transcriptional profiles of DNA damage from ciprofloxacin treated cells and the Δ*addA* mutant cells demonstrated a strong correlation (*r* ^2^ = 0.9) between their induced and repressed gene sets (Figure 1A,B). Indeed, 41 induced genes (Figure 1B) and 41 repressed genes identified by SAM were common to both profiles and this overlap was statistically significant (p<0.001, χ^2^ test), demonstrating that these two DNA damaging conditions regulated a similar subset of genes. We focused on the 41 genes induced in both ciprofloxacin treated cells and the Δ*addA* mutant (Figure 1B). No DNA repair genes, a hallmark of the SOS response, were induced in both conditions, but we were surprised to note several genes involved in natural competence for DNA transformation (explored further below). Our findings are similar to those in diverse species, which demonstrate that DNA repair genes are only one of many classes of genes regulated by DNA damage [25], [26]. Consistent with these prior studies, we found genes required for energy metabolism, membrane proteins, and fatty acid biosynthesis (Table S1) are regulated in response to DNA damage, although the contribution of these genes to survival in the face of DNA damage is not well understood in any species. Several cell division genes were also induced (*minE*, *ftsK*, *fic*); however there is no obvious homolog of the SOS-regulated cell division inhibitor *sulA* in *H. pylori* [33]. Interestingly, translation factors were also induced. Although induction of translation has not been observed as part of the DNA damage response in other bacterial species, we explore below its contribution to the DNA damage response. Finally, 30% of the induced genes are species-specific genes, which may function in cellular responses to DNA damage or have co-opted an existing regulatory pathway for their expression [34].

### RecA is required for the transcriptional response to DNA damage

RecA expression is often induced by DNA damage, thus increasing induction of SOS [25], [26], [35]. Although *H. pylori* seems to lack *lexA*, it seemed possible that RecA may be required for a transcriptional response to DNA damage. Thus we specifically queried the expression of RecA in response to DNA damage. Real-time quantitative PCR (qPCR) of either ciprofloxacin-treated wild-type cells or cells lacking *addA* revealed expression of *recA* was slightly repressed (Table 1). To test whether *recA* is required for the induction of DNA damage regulated genes, cDNA microarrays were used to measure transcriptional changes in cells lacking *recA* that were either untreated or treated with ciprofloxacin (Figure 1B). Only seven genes were induced in response to ciprofloxacin treatment in cells lacking *recA* and there was no overlap with the DNA damage responsive genes defined above. The absence of a transcriptional response in cells lacking *recA* suggests that RecA participates in sensing and transmission of the DNA damage signal, despite the absence of *lexA* in *H. pylori*.

**Table 1:** Transcription of the *com* T4SS is induced by DNA damage but *recA* expression is not.

Genotype tested	*recA*	*comB8*	*comB9*
Wild-type plus cipro	−1.9 (−1.1–−3.1)	3.5 (2.7–4.4)	4.3 (3.5–5.2)
Δ*addA*	−1.7 (−1.1–−2.9)	5.5 (4.3–7)	6.6 (4.9–8.9)
Δ*recA*	ND	1.2 (1.0–1.5)	1.4 (1.0–1.8)
Δ*recA* plus cipro	ND	ND	1.2 (1.0–1.3)
*comB4^IE^*	ND	ND	3.1 (2.7–3.6)
Δ*comB10 ΔaddA*	ND	1.6 (1.3–2)	2.1 (1.7–2.7)
Δ*comB10* plus cipro	ND	ND	1.1 (0.9–1.3)

The fold change in transcription (range) for the indicated gene was measured for cells of the indicated genotype by real-time PCR using the comparative method. ND: not done.

### Natural competence is induced by DNA damage, but not other cellular stresses

Gene-set analysis of gene ontology (GO) terms was used to further identify pathways undergoing transcription changes in response to ciprofloxacin treatment. As genes in the same GO classes are both induced and repressed during the DNA damage response in diverse organisms [25], [26] a bipartite signal may be expected for some gene sets. Thus, a statistic was calculated for each GO term based on the absolute value of fold-induction (Materials and Methods). Genetic exchange was among the terms significantly enriched in wild-type cells treated with ciprofloxacin (Table S3). The genetic exchange category includes several genes that comprise the *com* T4SS and are essential for natural transformation [19], [36]. Moreover, *com* T4SS components *comB3*, *comB4* and *comB9*, which reside in two separate operons, *comB2-4* and *comB6-10* [36], [37], are among the 41 genes significantly induced by DNA damage (Figure 1A,B). qPCR confirmed transcriptional induction of *comB8* and *comB9* in wild-type cells treated with ciprofloxacin but not in *ΔrecA* cells (Table 1).

**Figure 2:**
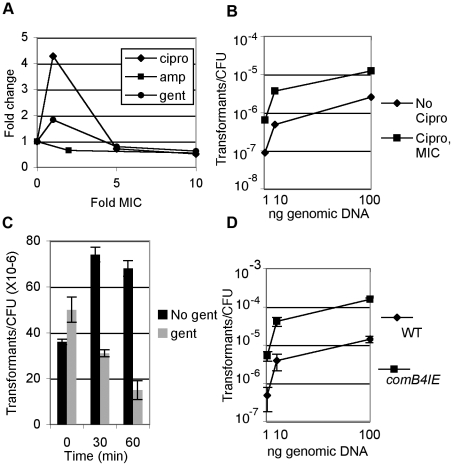
DNA damage increases natural transformation. **A.** Wild-type cells were treated at or above the MIC of ciprofloxacin (cipro), ampicillin (amp) (0.016 ug/ml [50]) or gentamicin (1 ug/ml, data not shown) for 2.5 hours and transformed with 20ng genomic DNA harboring an antibiotic resistance cassette and the fraction of cells transformed per CFU was determined. **B.** Wild-type cells were treated with the minimum inhibitory concentration (MIC) of ciprofloxacin for 2.5 hours and the fraction of cells transformed by genomic DNA per CFU was determined. Wild-type transformation frequency ranges between 10^−7^ and 10^−4^, depending on DNA concentration and experiment and for each panel, error bars are the standard deviation of the mean with at least three replicates for each point and a representative from two experiments is shown. **C.** Wild-type cells were treated with 10× MIC gentamicin for the indicated time and the fraction of cells transformed by genomic DNA per CFU was determined either by plating directly to selective medium or by plating to non-selective medium to allow translation of the selective marker prior to selection. **D.** Increased expression of *comB4* (*comB4^IE^*) increases natural transformation. The fraction of wild-type cells and *comB4^IE^* cells transformed by genomic DNA was determined.

Many organisms are competent only under certain environmental conditions, such as starvation [38]. In contrast, *H. pylori* is competent throughout logarithmic growth [21] and little is known about its regulation. Since expression of the *com* T4SS was DNA damage inducible, we tested whether natural transformation is increased by DNA damage. Wild-type cells were exposed to ciprofloxacin at increasing concentrations and the frequency of transformation with exogenously added genomic DNA harboring an antibiotic-resistance cassette was measured. Cells treated with the minimum inhibitory concentration of ciprofloxacin [39] had a 4–5 fold higher frequency of transformation than untreated cells (Figure 2A,B) and the frequency of transformation was easily saturated (Figure 2B). Further increasing the concentration of ciprofloxacin decreased transformation frequency, possibly due to higher levels of DNA damage (Figure 2A). No transformants were obtained after ciprofloxacin treatment of the Δ*comB10* mutant at any concentration, demonstrating that the ciprofloxacin-induced increase in natural transformation depends on activity of the *com* T4SS.

We also investigated whether other classes of antibiotics influence natural transformation. No increase in transformation was observed after treatment with ampicillin and there was little change in cells treated with gentamicin (Figure 2A). To further explore whether the slight increase in transformation observed with gentamicin at the minimal inhibitory concentration (MIC) resulted from weak induction of the DNA damage response, we queried the transcriptional response of *H. pylori* to gentamicin by microarray analysis. We observed induction of 80 genes (Table S4) and repression of 114 genes using a 1%FDR. Comparison of the gentamicin responsive genes to the DNA damage responsive genes showed an overlap of only 4 induced genes and 4 repressed genes, which was not statistically significant in either case (*p* = 0.2, χ^2^ test).

As translation factors are induced by DNA damage (Figure 1B), we determined whether ongoing translation is required for natural competence. Cells were pre-treated for various times with gentamicin at 10× MIC to fully block translation, then transformed with genomic DNA. Release of cells from gentamicin was required to recover expression of the antibiotic resistance cassette prior to selection (data not shown). One hour of pre-treatment with gentamicin caused a 5-fold reduction in transformation frequency (Figure 2C). Inhibition of transformation by gentamicin suggests that some component of the natural competence pathway must be continually synthesized and that transcriptional induction of translation by DNA damage is necessary for induction of natural competence. Taken together, these results show that induction of natural transformation is a specific response to DNA damaging agents probably mediated by increased transcription and translation of competence genes.

### DNA uptake sustains expression of DNA damage responsive genes

As mentioned above, previous work had suggested that competence was constitutive in *H. pylori*. To explore whether a component of the natural transformation machinery is limiting for competence, we tested whether expression of a single *com* component might increase transformation. Merodiploids with increased expression of competence apparatus components, *comB10* and *dprA*, a cytosolic protein required for competence, showed no increase in transformation frequency (data not shown); however two independent clones of the *comB4* merodiploid (*comB4^IE^* for increased expression) were found to have a 5–8 fold increased transformation frequency (Figure 2D).

**Figure 3:**
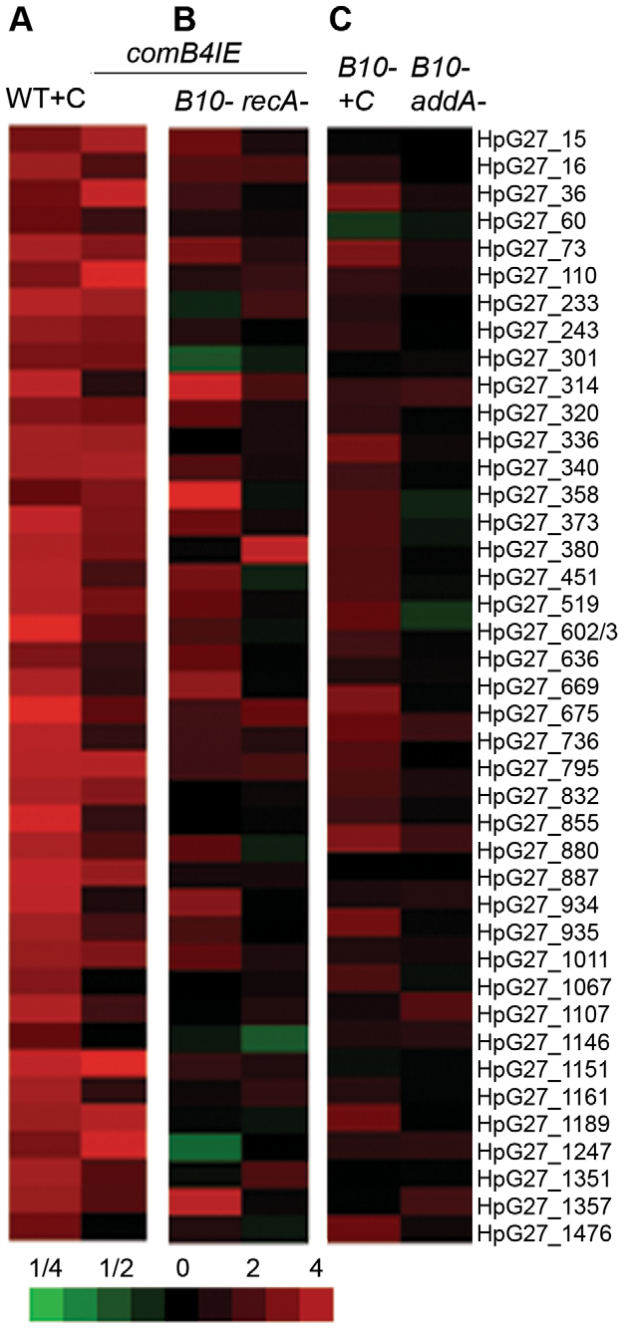
*com* T4SS control of the DNA damage response. **A.** The *comB4* merodiploid (*comB4^IE^*) induces expression of DNA damage responsive genes. Untreated wild-type cells are compared to either wild-type cells treated with ciprofloxacin (WT+C) or untreated *comB4^IE^* cells and the mean of fold change in RNA expression measured by microarray from three independent cultures is shown. Bottom, scale bar indicates fold change for heat maps in A,B,C. **B.** Increased expression of DNA damage responsive genes in *comB4^IE^* cells requires *recA* and the *com* T4SS. Untreated wild-type cells are compared to either *comB4^IE^ ΔcomB10* (*comB4^IE^ B10*) cells, or *comB4^IE^ ΔrecA* cells and data is represented as in Figure 1A. **C.** Full induction of DNA damage responsive genes by DNA damage requires the *com* T4SS. Untreated wild-type cells are compared to ciprofloxacin treated Δ*comB10* mutant cells (*B10-* +C) and untreated Δ*addA* Δ*comB10* double mutant cells (*B10- addA-*). Data is represented as in Figure 1A.

ComB4 is the only known ATPase of the Com T4SS and is thought to drive translocation of DNA through the competence machinery [19]. Increased competence in *comB4^IE^* cells could result from either increased activity of the ComB4 ATPase or increased expression of the whole *com* T4SS. qPCR of *comB9* showed that its transcription was increased in *comB4^IE^* cells (Table 1) prompting us to perform microarray analysis of *comB4^IE^* cells. Although no genes were significantly repressed, there were 167 genes significantly induced (SAM 5% FDR) and 57 overlapped with the induced gene set for ciprofloxacin, which was statistically significant (p<0.001, χ^2^ test) (Table S5). In addition, SAM analysis demonstrated that 25 of the 41 genes regulated by DNA damage were induced, including *com* T4SS components *comB3* (*HpG27_15*) and *comB9* (*HpG27_36*) (Figure 3A) further suggesting activation of at least a subset of the DNA damage induced transcriptional program. The *comB4^IE^* cells showed similar sensitivity to DNA damaging agents and an equivalent mutation rate to wild-type cells (Table 2), suggesting these cell do not accumulate unrepaired DNA damage. Thus, increased expression of *comB4* produces a similar effect as ciprofloxacin treatment and the Δ*addA* mutant, although it does not appear to cause DNA damage.

**Table 2:** Mutation rate and sensitivity to DNA damage are unchanged in cells expressing the DNA damage response.

Genotype	mutants/cell/generation (95% CI)	MIC ciprofloxacin, µg/ml (+/− SD)
Wild type	4.7×10^−9^ (2.7–7.1×10^−9^)	0.18 (+/−0.03)
Δ*addA*	1.5×10^−8^ (0.5–8.4×10−8)*	0.053 (+/−.01)
*comB4^IE^*	4.5×10^−9^ (1.6–5.4×10^−9^)**	0.17 (+/−0.03)

Mutation rate of *sacB* was determined by fluctuation analysis and p values were determined by student's T-test in comparison to wild type. *p = 0.07, **p = 1. MIC of ciprofloxacin was determined by E-test (Biodisk). SD = standard deviation.

We next defined the requirements for transcriptional induction of DNA damage responsive genes in *comB4^IE^*. *recA* was required for induction (Figure 3B), suggesting that transcriptional induction of the DNA damage responsive genes in *comB4IE* occurs through a similar pathway as in ciprofloxacin treated cells. We hypothesized that increased DNA uptake in *comB4IE* cells is sensed by RecA and leads to transcriptional induction of the DNA damage responsive gene set. In support of this hypothesis, blocking DNA uptake by mutation of *comB10* significantly decreases transcriptional induction of DNA damage responsive genes in the *comB4^IE^* cells (Figure 3B). Comparison of the *comB4^IE^* transcriptional profile with either the *comB4^IE^* Δ*comB10* mutant or the *comB4^IE^* Δ*recA* mutant profile showed no statistically significant associations (χ^2^ test, *p* = 0.2). One possible explanation for these findings is that increased DNA uptake induces DNA damage responsive genes. Alternatively, a component of the natural competence machinery may act as a transcriptional regulator of the DNA damage responsive genes.

To further support the role for DNA uptake in stimulating the DNA damage response, we tested whether natural competence is required to stimulate transcription in cells undergoing DNA damage. In the Δ*addA ΔcomB10* double mutant and the *ΔcomB10* mutant treated with ciprofloxacin, qPCR revealed that *comB9* was not induced (Table 1). Microarray analysis revealed no transcriptional changes in the Δ*comB10* single mutant compared to wild-type cells (data not shown) and no induction of DNA damage responsive genes in the *ΔaddA ΔcomB10* double mutant (Figure 3C). In the Δ*comB10* mutant treated with ciprofloxacin, only 4 of 41 DNA damage responsive genes were significantly induced (SAM 5% FDR), but close inspection of the microarray data indicated that the DNA damage response was weakly induced (e.g. HPG27_36, HPG27_73, Figure 3C), suggesting a role for natural competence in sustaining expression of DNA damage responsive genes.

### A phage lysozyme-like gene contributes to DNA donation

**Figure 4:**
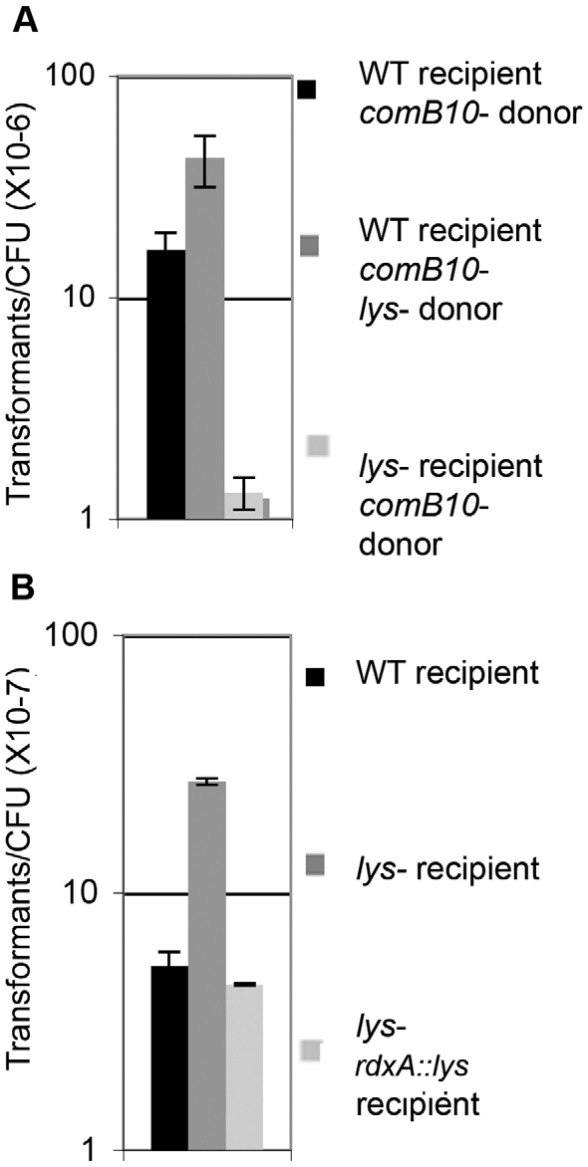
Cells lacking lysozyme take up more genomic DNA than wild-type, but are less able to acquire DNA from donor cells. **A.** Log-phase recipient cells were mixed with stationary phase donor cells and the frequency of transformation was determined. **B.** The fraction of cells of the indicated genotype transformed by genomic DNA per CFU was determined using 10 ng genomic DNA. Error bars are the standard deviation of the mean with at least three replicates for each point and a representative of two independent experiments is shown.

**Figure 5:**
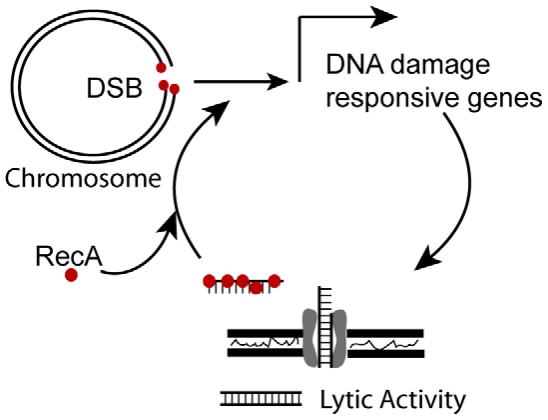
Model for positive feedback of DNA on DNA damage responsive genes. RecA binds DSBs and transcription of DNA damage responsive genes is induced that includes a lysozyme-like protein, which may be used to acquire DNA and the *com* T4SS, which transports DNA in the cell. Once in the cytosol, transforming DNA is bound by RecA and further induces transcription of DNA damage responsive genes.

Since no exogenous DNA was added to the transcriptional profiling experiments, cells in culture are the likely source of DNA taken up by the *com* T4SS [8]. The rate of genetic exchange between Δ*comB10* mutant (donor) and wild-type (recipient) cells was measured by fluctuation analysis. Genetic exchange of a single chromosomally encoded antibiotic resistance gene occurred at a rate of 2.4–5.8×10^−10^ exchanges/cell/generation, suggesting that cells are constantly exposed to free DNA liberated from other cells in the culture. Closer inspection of the genes regulated by DNA damage revealed an induced gene (*lys*, HPG27_320) that is homologous to phage T4 lysozyme and has demonstrated lysozyme activity [40]. We hypothesized that this protein lyses neighboring cells, thus liberating DNA for uptake during culture. To test this idea, we used stationary phase cells that were non-competent (the *ΔcomB10* mutant) as donor cells so that genetic exchange is unidirectional. Logarithmically growing wild-type cells (recipient) showed a 12-fold higher transformation efficiency than the Δ*lys* mutant recipient (Figure 4A), indicating that the lysozyme expressing cells could obtain more DNA from donor cells for transformation. Moreover, the Δ*lys* mutant is transformed with purified genomic DNA at the same or higher frequency than wild-type cells (Figure 4B), indicating that *Lys* is not required for transformation. These results suggest that a DNA damage-induced lysozyme may target susceptible cells in culture and provide a source of DNA for uptake. DNA uptake then activates the DNA damage responsive genes in a positive feedback loop (Figure 5).

### The *H. pylori* DNA damage response does not affect mutation rate

GO analysis gave no indication of induction of DNA repair functions by DNA damage (Table S3), but many of DNA damage responsive genes are not annotated and might have been missed. Since the *ΔcomB10* mutant does not induce DNA damage responsive genes, we investigated whether the Δ*comB10* mutant is sensitive to DNA damaging agents, but sensitivity to ciprofloxacin and mutation rate were indistinguishable from wild-type cells (Table 2). In *E. coli*, mutation rates are increased by the SOS response through induction of error prone polymerases [24], therefore we determined the mutation rate for *H. pylori* cells that constitutively induce DNA damage responsive genes. The *comB4^IE^* cells have a mutation rate equivalent to wild-type cells, whereas the repair-defective Δ*addA* cells have a rate that is only slightly elevated and is not statistically distinguishable from wild type (Table 2). Thus, even under stressful conditions this *H. pylori* strain maintains a low mutation rate, which further supports the hypothesis that *H. pylori* strain variation is driven by recombination among diverse strains [14], [15].

### Natural competence can be detrimental during stomach colonization

**Figure 6:**
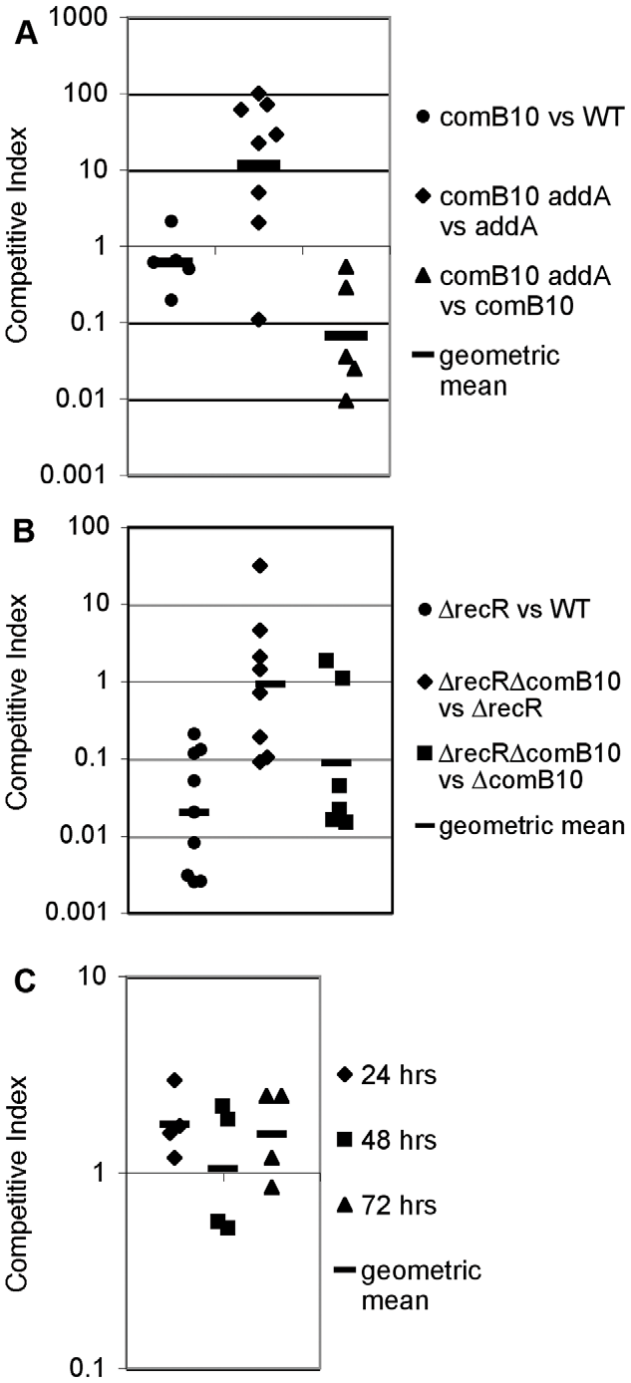
DNA damage responsive genes do not contribute to DNA repair during stomach colonization. Each data point shows the competitive index of mutant cells vs wild-type cells or the indicated double mutant compared to either single mutant for a single mouse after one-week stomach colonization (A, B) or for a single well during co-culture in broth (C) and bars indicate the geometric mean. **A.** Competition between the Δ*comB10* mutant and wild-type cells shows no defect during stomach colonization; however stomach colonization of the Δ*addA* mutant is improved by disruption of natural competence. **B.** Competition between the Δ*recR* mutant and wild-type cells shows a strong defect during stomach colonization, but is unaffected by disruption of natural competence. **C.** Competence does not affect growth of the Δ*addA* mutant in broth culture. The Δ*addA* Δ*comB10* double mutant and the Δ*addA* mutant were maintained in logarithmic growth for three days in broth culture by dilution.

Our results suggest that competence is a major output of the DNA damage response in *H. pylori*, but does not contribute to DNA repair or mutation. A mouse colonization assay [41] was used to further explore the relationship between DNA damage and competence during infection. The Δ*comB10* mutant showed equivalent colonization to wild-type cells in a competition assay in which differentially marked mutant and wild-type cells were introduced into the mouse stomach for one week (Figure 6A). In contrast, the Δ*addA* mutant cells are compromised for infection in both competition and single strain infections [22]. Since the Δ*addA* mutant can colonize, albeit with lower efficiency than wild-type cells, we tested whether the *comB10* mutation would further compromise the Δ*addA* mutant colonization by virtue of its requirement for activation of DNA damage responsive genes and competence. As shown in Figure 6A, the opposite result was observed: the Δ*comB10 ΔaddA* double mutant showed enhanced colonization relative to the Δ*addA* mutant, but not the Δ*comB10* mutant. We tested whether this effect is specific to cells lacking DSB repair. The Δ*recR* mutant, which lacks single strand break repair, is defective for stomach colonization, but this defect is not suppressed by the loss of *com* T4SS activity (Figure 6B). The enhancement of growth by loss of *com* T4SS activity was not observed in broth culture. Although the Δ*addA* mutant grows slower than wild-type cells in broth culture (Figure S1), the Δ*addA* mutant and the Δ*addA ΔcomB10* double mutant grew at the same rate (Figure 6C). In total, our results suggest that neither competence nor DNA damage responsive genes contribute significantly to DNA repair during culture or initial stomach colonization. Furthermore, during colonization, the *com* T4SS exerts a fitness cost in the context of a DNA repair mutant. Thus, the observed transcriptional and translational control over natural competence may represent mechanisms to control a costly process during colonization.

### Conclusions

Our data reveal a connection between natural competence and the response to DNA damage in *H. pylori*. Similar to our observations in *H. pylori*, natural competence is induced by DNA damage and other stresses in the Gram-positive organism *S. pneumoniae* [29]. In contrast, *S. pneumoniae* regulation of competence and its molecular machinery for DNA uptake are completely different from *H. pylori* [19], [38], suggesting induction of competence in response to DNA damage is the product of convergent evolution. In *H. pylori* our data support a model (Figure 5) whereby DNA damage induces RecA-dependent expression of both a lysozyme-like protein, which stimulates donation of DNA from susceptible *H. pylori*, and the *com* T4SS, which increases the import of foreign DNA. Through a second RecA-dependent mechanism, DNA acquired via the *com* T4SS induces DNA damage responsive genes, thus amplifying the signal. A similar mechanism for signal amplification occurs in eukaryotic cells in which processing of DNA breaks creates single stranded DNA oligonucleotides that trigger the DNA damage checkpoint [42]. This newly described connection between the DNA damage response and DNA uptake suggests that natural competence contributes to persistence of *H. pylori* in its human host and explains its retention in most clinical isolates [18]. As patients are sometimes infected with more than one distinct strain [12], [13], [14], [15], up-regulation of natural competence may increase exchange of antibiotic resistance alleles and facilitate selection of fitter variants through re-assortment of pre-existing alleles. Our study suggests that *H. pylori* have co-opted signals of their harsh environment, namely DNA damage and extracellular DNA to induce genetic exchange within a heterogeneous population.

## Materials and Methods

### Ethics statement

All animal studies were done under practices and procedures of Animal Biosafety Level 2. The facility is fully accredited by the Association for Assessment and Accreditation of Laboratory Animal Care, International. The FHCRC Institutional Animal Care and Use Committee approved all activities.

### Media and antibiotics

*H. pylori* strains were grown on solid horse blood agar (HB) plates containing 4% Columbia agar base (BD Bioscience), 5% defibrinated horse blood (HemoStat Laboratories), 0.2% β-cyclodextrin (Sigma), vancomycin (Sigma; 10 µg ml−1), cefsulodin (Sigma; 5 µg ml−1), polymyxin B (Sigma; 2.5 U ml−1), trimethoprim (Sigma; 5 µg ml−1) and amphotericin B (Sigma; 8 µg ml−1) at 37°C either under a microaerobic atmosphere generated using a CampyGen sachet (Oxoid) in a gas pack jar or in an incubator equilibrated with 14% CO2 and 86% air. For liquid culture, H. pylori was grown in Brucella broth (BD Biosciences) containing 10% fetal bovine serum (BB10; Hyclone) with shaking in a gas pack jar with a CampyGen sachet. For antibiotic resistance marker selection, bacterial media were additionally supplemented with kanamycin (50 µg ml−1), chloramphenicol (Cm; 15 µg ml−1) or metronidazole (Mtz; 36 µg ml−1). When culturing bacteria from mouse stomachs, Bacitracin (Bac; 200 µg ml−1) was added to eliminate contamination. For cDNA microarray and natural transformation assays BB10 medium was supplemented with ciprofloxacin, ampicillin, or gentamicin as indicated (Sigma).

### Strains and plasmids

All *H. pylori* isogenic mutants were generated as described [23] in strain NSH57 [43]. Strains are listed in Table S6 and oligonucleotides in Table S7. All complementation constructs were generated and introduced into *H. pylori* as described [23].

### Antibiotic resistance testing

*H. pylori* were grown overnight in liquid culture to optical density at 600 nm (OD_600_) 0.3 and 200 µL was plated on solid medium lacking all other antimicrobials, incubated for 30 minutes in a CO_2_ incubator. E-test strips (AB Biodisk) were then placed on the plates, which were further incubated for 2 days and read according to the manufacturers instructions.

### RNA isolation and DNA microarray analysis

An overnight liquid culture was grown to (OD_600_) 0.8, then collected on 0.1 µm pore size filters (Whatman) and frozen in liquid nitrogen. RNA was extracted as described [44]. Approximately 10 µg RNA was reverse transcribed with Superscript II (Invitrogen), 1.5 mM each dATP, dCTP, dGTP, 0.75 mM each dTTP, 5-(3-aminoallyl)-2′-deoxyuridine-5′-triophosphate, (aa-dUTP) and random octamer primers (Fisher). To hydrolyze RNA, 100 mM EDTA, 200 mM NaOH was added and the mixture was heated to 65°C for 15 minutes. cDNA was purified over DNA Clean and Concentrator-5 (Zymoresearch), eluted with 50 mM sodium bicarbonate and coupled to Cy3- (untreated sample) or Cy5- (treated sample) mono NSH ester (Amersham) for one hour at room temperature. Treated and untreated samples were then mixed and unincorporated dye removed over a DNA Clean and Concentrator-5 (Zymoresearch), with samples eluted into 10 mM Tris-HCl and prepared for hybridization to custom DNA microarrays as described [45].

Microarray scanning and analysis were performed on a GenePix 4000B scanner (Axon) using GenePix Pro 6.0 software (Axon). Spots were filtered for slide abnormalities and signal from duplicate spots were averaged. These data were stored and processed in the Stanford Microarray database (http://smd.stanford.edu/). Values for genes found in strain G27 by comparative genomic hybridization [15], [46] were extracted from this set and filtered for a regression correlation >0.6 of the Log_2_ red/green normalized ratio (mean). These data sets were then either analyzed using SAM [31] or clustered using Cluster and visualized with Treeview (http://rana.lbl.gov/EisenSoftware.htm). To determine whether the overlap between arrays was significant, we used a chi-squared test comparing the induced genes in both conditions to the repressed or unchanged genes for each condition. Raw microarray data have been deposited in NCBI's Gene Expression Omnibus and are accessible through GEO Series accession number GSE19334 (http://www.ncbi.nlm.nih.gov/geo/query/acc.cgi?acc=GSE19334).

### Creation of a gene ontology for *H. pylori* and gene set analysis

Two gene ontologies were created for *H. pylori*. GO terms associated with *H. pylori* strain 26695 were downloaded from the Uni-prot database (http://www.uniprot.org/). We then hand-annotated genes that were not identified on this database using GO terms from Amigo (amigo.geneontology.org). *H. pylori*-specific and conserved hypothetical genes were identified using genolist.pasteur.fr/PyloriGene/ and GO terms were created for these categories. This procedure produced the generation0 GO list. In addition, the Bioconductor GO.db was used to identify the parent each of those GO terms, to provide a more general set of associations for each gene and creating the generation1 gene ontology.

Each array was normalized individually to adjust values for dye effects and background-corrected in the red (treated) and green (untreated) channel data. Array effects were normalized across 10 microarrays from ciprofloxacin treated cells, *comB4* ^IE^ cells and the Δ*addA* mutant. Expression was calculated as the difference of the normalized log_2_ ratio of the red and green channels in each array. Finally, the two or four values for each probe on an array were averaged. We used the R package ‘limma’ to estimate treatment effects on expression.

A GSEA-style approach was used to assess differential expression for each of GO term with 10 or more associated probes [47]. A statistic was generated by summing the absolute value of the test t-statistics of probes associated with a GO term. A permutation test was used to estimate the p-value since the analytical function describing this distribution is not known.

### qPCR

RNA was extracted as described above and reverse transcribed in a standard reaction with Superscript II (Invitrogen). qPCR was performed in a standard reaction using SYBR green on an ABI prism 7900HT sequence detection system (Applied Biosystems). Expression differences were calculated using the ΔΔCT method.

### Quantification of natural transformation

Cells of the indicated genotype were grown overnight to approximately OD_600_ 0.9 in shaking culture, then placed in 96 well plates (6×10^7^ cells/well), and the indicated amount of genomic DNA harboring the *aph3* gene, which confers kanamycin resistance, at a neutral locus [48] was added. After one hour, appropriate dilutions were plated to non-selective medium to determine the number of colony forming units. In addition, 50 µL was plated to non-selective plates, incubated for 24 hours to allow expression of the antibiotic resistance cassette, then replica plated to selective medium to determine the frequency of transformation. In some cases, cells were directly plated to selective medium, as described in the text.

To determine the frequency of transformation using stationary phase cells as donors, the recipient cells were grown overnight to log phase. Donor cells were grown to OD_600_ = 2 and then incubated for a further 16 hours. Donor and recipient cells were mixed 1∶1 for three hours, then plated to the appropriate selective medium to determine the frequency of transformation of the recipient cells to the donor genotype.

### Competition in broth culture

Cells were grown overnight in liquid culture to mid-log phase, diluted to OD_600_ = 0.0015 for each clone and grown 24 hours in 96 well plates. After 24 and 48 hours, cells were diluted 450-fold into fresh medium and incubated for another 24 hours. At each time point, cells were titred for colony forming units (CFU) on selective medium and non-selective medium to determine the ratio of each clone in the mixture. The competitive index was determined by dividing the CFU ratio of the two clones at each time point by the starting ratio.

### Mutation rate and rate of exchange between cells in culture

Mutation rate was measured in cells harboring a dual cassette consisting of *aph3*, which confers kanamycin resistance and *sacB*, which confers sensitivity to sucrose that was integrated at the *omp27* locus (for wild-type and *comB4IE* cells) and at the *rdxA* locus (for wild-type and *ΔaddA* cells). Cells were grown overnight in liquid culture to mid-log phase, diluted to 10 cells/200µL in BB10 in 20 wells of a 96 well plate and incubated for 72 hours or 96 hours (for the Δ*addA* mutant). The entire well was plated to medium containing sucrose and 4 wells were titred on non-selective medium to determine average cell number. The mutation rate was calculated using the maximum likelihood method. To determine the rate of exchange between cells in culture, cells were similarly diluted and then mixed together. Wild-type cells were marked with the *aph3* gene at a neutral locus [48] and the DNA donor, *ΔcomB10::cat*, is resistant to chloramphenicol. The entire well was plated to medium containing kanamycin and chloramphenicol. The rate of exchange was calculated using the maximum likelihood method [49].

### Mouse colonization

Female C57BL/6 mice 24–28 days old were obtained from Charles River Laboratories and certified free of endogenous *Helicobacter* infection by the vendor. The mice were housed in sterilized microisolator cages with irradiated PMI 5053 rodent chow, autoclaved corn cob bedding, and acidified, reverse-osmosis purified water provided ad libitum. All mouse colonization experiments were performed exactly as described [23].

## Supporting Information

Figure S1 The *ΔaddA* mutant shows decreased replication efficiency in broth culture. The *ΔaddA* mutant and wild-type cells were maintained in logarithmic growth for three days in broth culture by dilution. B. The *ΔrecR* mutant shows no change in replication efficiency in broth culture.
(0.19 MB TIF)

Table S1 Similar genes are induced in cells treated with ciprofloxacin and in the *ΔaddA* mutant. All genes listed are significantly induced by SAM, using a 1% FDR for ciprofloxacin and a 5% FDR for the *ΔaddA* mutant. DNA damage regulon genes are highlighted in bold. Induced transcripts are listed in genome order for the strain G27 [46].
(0.22 MB DOC)

Table S2 The *ΔrecR* mutant shows no significant transcriptional induction, although there are genes showing greater than 1.6-fold induction by microarray in the *ΔrecR* mutant relative to wild-type cells. No genes listed are significantly induced by SAM at 1% FDR and no FDR below 85% generates significant changes from wild-type cells. Independent clones of the *ΔrecR* mutant marked with different antibiotic resistance cassettes gave similar transcriptional profiles.
(0.20 MB DOC)

Table S3 Gene set analysis of gene ontology (GO) terms for ciprofloxacin treated cells. Terms listed have p<0.03, using the generation1 gene ontology (Materials and Methods).
(0.05 MB DOC)

Table S4 Genes significantly induced in wild-type cells treated with gentamicin (SAM, 1% FDR). DNA damage regulon genes are highlighted in bold. Induced transcripts are listed in genome order for the strain G27 [46].
(0.11 MB DOC)

Table S5 Genes significantly induced in *comB4IE* cells (SAM, 1% FDR). DNA damage regulon genes are highlighted in bold. Induced transcripts are listed in genome order for the strain G27 [46].
(0.21 MB DOC)

Table S6 *H. pylori* strains used in these studies *H. pylori* strains used in these studies
(0.03 MB DOC)

Table S7 Oligonucleotides used in this study. Gene specific sequences are in upper case and sequences added for cloning in lower case. *Denotes oligos used for qPCR. All others were used to generate deletions # denotes oligos also used to generate complementation plasmids.
(0.05 MB DOC)

## References

[1] Peek RM, Blaser MJ (2002). *Helicobacter pylori* and gastrointestinal tract adenocarcinomas.. Nat Rev Cancer.

[2] Chey WD, Wong BC (2007). American College of Gastroenterology guideline on the management of *Helicobacter pylori* infection.. Am J Gastroenterol.

[3] Graham DY (2009). Efficient identification and evaluation of effective *Helicobacter pylori* therapies.. Clin Gastroenterol Hepatol.

[4] Suerbaum S, Smith JM, Bapumia K, Morelli G, Smith NH (1998). Free recombination within *Helicobacter pylori*.. Proc Natl Acad Sci U S A.

[5] Alm RA, Ling LS, Moir DT, King BL, Brown ED (1999). Genomic-sequence comparison of two unrelated isolates of the human gastric pathogen *Helicobacter pylori*.. Nature.

[6] Gressmann H, Linz B, Ghai R, Pleissner KP, Schlapbach R (2005). Gain and loss of multiple genes during the evolution of *Helicobacter pylori*.. PLoS Genet.

[7] Salama N, Guillemin K, McDaniel TK, Sherlock G, Tompkins L (2000). A whole-genome microarray reveals genetic diversity among *Helicobacter pylori* strains.. Proc Natl Acad Sci U S A.

[8] Baltrus DA, Guillemin K, Phillips PC (2008). Natural transformation increases the rate of adaptation in the human pathogen *Helicobacter pylori*.. Evolution.

[9] Luria SE, Delbruck M (1943). Mutations of Bacteria from Virus Sensitivity to Virus Resistance.. Genetics.

[10] Björkholm B, Sjolund M, Falk PG, Berg OG, Engstrand L (2001). Mutation frequency and biological cost of antibiotic resistance in *Helicobacter pylori*.. Proc Natl Acad Sci U S A.

[11] Kang JM, Iovine NM, Blaser MJ (2006). A paradigm for direct stress-induced mutation in prokaryotes.. FASEB J.

[12] Taylor NS, Fox JG, Akopyants NS, Berg DE, Thompson N (1995). Long-term colonization with single and multiple strains of Helicobacter pylori assessed by DNA fingerprinting.. J Clin Microbiol.

[13] Berg DE, Gilman RH, Lelwala-Guruge J, Srivastava K, Valdez Y (1997). *Helicobacter pylori* populations in Peruvian patients.. Clin Infect Dis.

[14] Schwarz S, Morelli G, Kusecek B, Manica A, Balloux F (2008). Horizontal versus familial transmission of *Helicobacter pylori*.. PLoS Pathog.

[15] Salama NR, Gonzalez-Valencia G, Deatherage B, Aviles-Jimenez F, Atherton JC (2007). Genetic analysis of *Helicobacter pylori* strain populations colonizing the stomach at different times postinfection.. J Bacteriol.

[16] Talarico S, Gold BD, Fero J, Thompson DT, Guarner J (2009). Pediatric *Helicobacter pylori* isolates display distinct gene coding capacities and virulence gene marker profiles.. J Clin Microbiol.

[17] Israel DA, Salama N, Krishna U, Rieger UM, Atherton JC (2001). *Helicobacter pylori* genetic diversity within the gastric niche of a single human host.. Proc Natl Acad Sci U S A.

[18] Yeh YC, Chang KC, Yang JC, Fang CT, Wang JT (2002). Association of metronidazole resistance and natural competence in *Helicobacter pylori*.. Antimicrob Agents Chemother.

[19] Hofreuter D, Odenbreit S, Haas R (2001). Natural transformation competence in *Helicobacter pylori* is mediated by the basic components of a type IV secretion system.. Mol Microbiol.

[20] Alvarez-Martinez CE, Christie PJ (2009). Biological diversity of prokaryotic type IV secretion systems.. Microbiol Mol Biol Rev.

[21] Baltrus DA, Guillemin K (2006). Multiple phases of competence occur during the *Helicobacter pylori* growth cycle.. FEMS Microbiol Lett.

[22] Amundsen SK, Fero J, Hansen LM, Cromie GA, Solnick JV (2008). *Helicobacter pylori* AddAB helicase-nuclease and RecA promote recombination-related DNA repair and survival during stomach colonization.. Mol Microbiol.

[23] Amundsen SK, Fero J, Salama NR, Smith GR (2009). Dual Nuclease and Helicase Activities of *Helicobacter pylori* AddAB Are Required for DNA Repair, Recombination, and Mouse Infectivity.. J Biol Chem.

[24] Galhardo RS, Hastings PJ, Rosenberg SM (2007). Mutation as a stress response and the regulation of evolvability.. Crit Rev Biochem Mol Biol.

[25] Cirz RT, Jones MB, Gingles NA, Minogue TD, Jarrahi B (2007). Complete and SOS-mediated response of *Staphylococcus aureus* to the antibiotic ciprofloxacin.. J Bacteriol.

[26] Cirz RT, O'Neill BM, Hammond JA, Head SR, Romesberg FE (2006). Defining the *Pseudomonas aeruginosa* SOS response and its role in the global response to the antibiotic ciprofloxacin.. J Bacteriol.

[27] Tomb JF, White O, Kerlavage AR, Clayton RA, Sutton GG (1997). The complete genome sequence of the gastric pathogen *Helicobacter pylori* [see comments] [published erratum appears in Nature 1997 Sep 25;389(6649):412].. Nature.

[28] Han J, Sahin O, Barton YW, Zhang Q (2008). Key role of Mfd in the development of fluoroquinolone resistance in *Campylobacter jejuni*.. PLoS Pathog.

[29] Prudhomme M, Attaiech L, Sanchez G, Martin B, Claverys JP (2006). Antibiotic stress induces genetic transformability in the human pathogen *Streptococcus pneumoniae*.. Science.

[30] Moore RA, Beckthold B, Wong S, Kureishi A, Bryan LE (1995). Nucleotide sequence of the *gyrA* gene and characterization of ciprofloxacin-resistant mutants of *Helicobacter pylori*.. Antimicrob Agents Chemother.

[31] Tusher VG, Tibshirani R, Chu G (2001). Significance analysis of microarrays applied to the ionizing radiation response.. Proc Natl Acad Sci U S A.

[32] Marsin S, Mathieu A, Kortulewski T, Guerois R, Radicella JP (2008). Unveiling novel RecO distant orthologues involved in homologous recombination.. PLoS Genet.

[33] Miyagishima SY, Wolk CP, Osteryoung KW (2005). Identification of cyanobacterial cell division genes by comparative and mutational analyses.. Mol Microbiol.

[34] Perez JC, Groisman EA (2009). Evolution of transcriptional regulatory circuits in bacteria.. Cell.

[35] da Rocha RP, Paquola AC, Marques Mdo V, Menck CF, Galhardo RS (2008). Characterization of the SOS regulon of *Caulobacter crescentus*.. J Bacteriol.

[36] Karnholz A, Hoefler C, Odenbreit S, Fischer W, Hofreuter D (2006). Functional and topological characterization of novel components of the *comB* DNA transformation competence system in *Helicobacter pylori*.. J Bacteriol.

[37] Sharma CM, Hoffmann S, Darfeuille F, Reignier J, Findeiss S The primary transcriptome of the major human pathogen *Helicobacter pylori.*. Nature.

[38] Claverys JP, Prudhomme M, Martin B (2006). Induction of competence regulons as a general response to stress in gram-positive bacteria.. Annu Rev Microbiol.

[39] Kang J, Huang S, Blaser MJ (2005). Structural and functional divergence of MutS2 from bacterial MutS1 and eukaryotic MSH4-MSH5 homologs.. J Bacteriol.

[40] Marsich E, Zuccato P, Rizzi S, Vetere A, Tonin E (2002). *Helicobacter pylori* expresses an autolytic enzyme: gene identification, cloning, and theoretical protein structure.. J Bacteriol.

[41] Salama NR, Otto G, Tompkins L, Falkow S (2001). Vacuolating cytotoxin of *Helicobacter pylori* plays a role during colonization in a mouse model of infection.. Infect Immun.

[42] Jazayeri A, Balestrini A, Garner E, Haber JE, Costanzo V (2008). Mre11-Rad50-Nbs1-dependent processing of DNA breaks generates oligonucleotides that stimulate ATM activity.. EMBO J.

[43] Baldwin DN, Shepherd B, Kraemer P, Hall MK, Sycuro LK (2007). Identification of *Helicobacter pylori* genes that contribute to stomach colonization.. Infect Immun.

[44] Merrell DS, Thompson LJ, Kim CC, Mitchell H, Tompkins LS (2003). Growth phase-dependent response of *Helicobacter pylori* to iron starvation.. Infect Immun.

[45] Thompson LJ, Merrell DS, Neilan BA, Mitchell H, Lee A (2003). Gene expression profiling of *Helicobacter pylori* reveals a growth-phase-dependent switch in virulence gene expression.. Infect Immun.

[46] Baltrus DA, Amieva MR, Covacci A, Lowe TM, Merrell DS (2008). The Complete Genome Sequence of *Helicobacter pylori* strain G27.. J Bacteriol.

[47] Hahne F, Huber W, Gentleman R, Falcon S (2008). Bioconductor Case Studies:.

[48] Langford ML, Zabaleta J, Ochoa AC, Testerman TL, McGee DJ (2006). In vitro and in vivo complementation of the *Helicobacter pylori* arginase mutant using an intergenic chromosomal site.. Helicobacter.

[49] Rosche WA, Foster PL (2000). Determining mutation rates in bacterial populations.. Methods.

[50] Hachem CY, Clarridge JE, Reddy R, Flamm R, Evans DG (1996). Antimicrobial susceptibility testing of *Helicobacter pylori*. Comparison of E-test, broth microdilution, and disk diffusion for ampicillin, clarithromycin, and metronidazole.. Diagn Microbiol Infect Dis.

