# Supplementary figures and images for: DNA Damage Triggers Genetic Exchange in Helicobacter pylori
Source: PLoS Pathog. 2010 Jul 29;6(7):e1001026. doi: 10.1371/journal.ppat.1001026 (PMC2912397; doi:10.1371/journal.ppat.1001026)

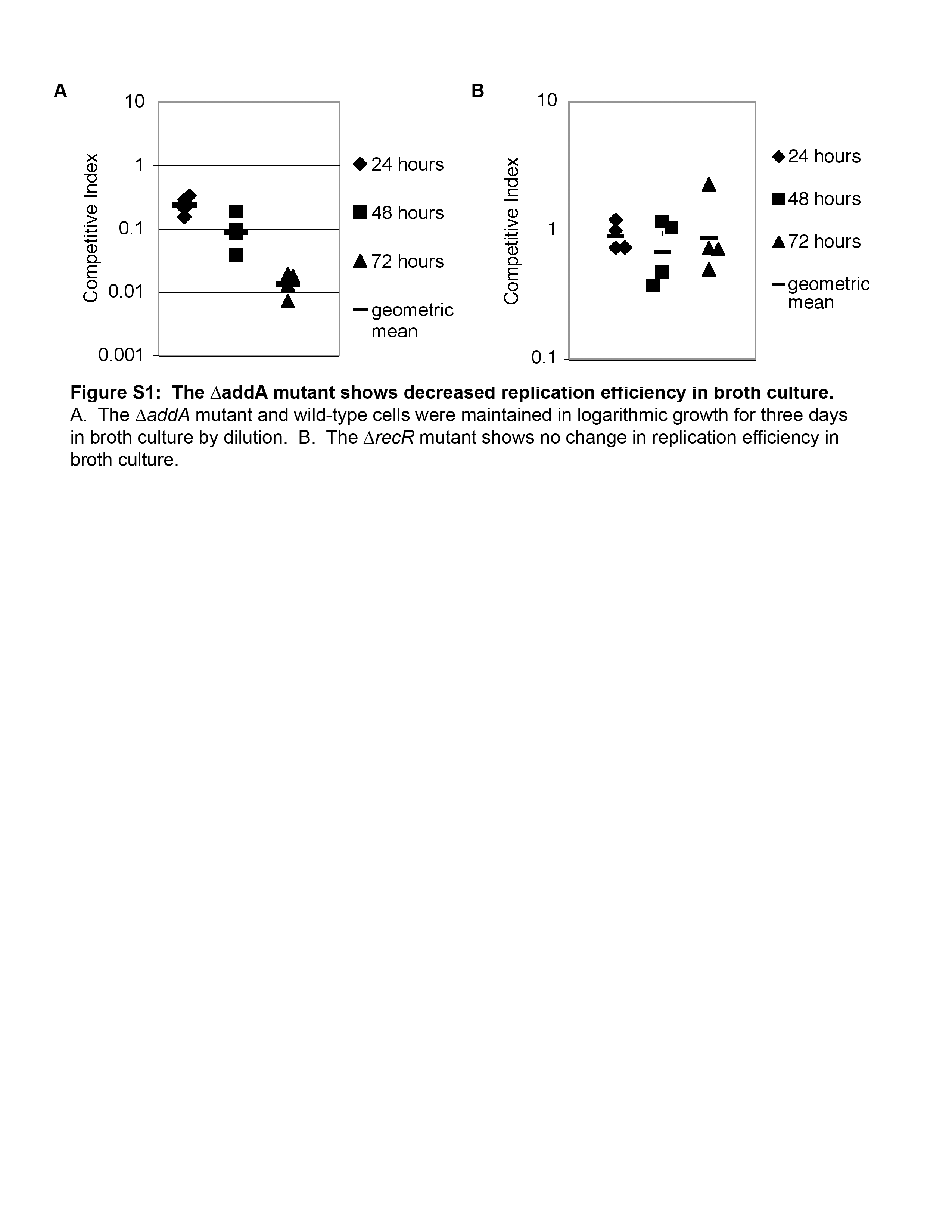

Supplement: Figure S1 — The ΔaddA mutant shows decreased replication efficiency in broth culture. The ΔaddA mutant and wild-type cells were maintained in logarithmic growth for three days in broth culture by dilution. B. The ΔrecR mutant shows no change in replication efficiency in broth culture. (0.19 MB TIF) [file ppat.1001026.s001.tif]
