# Supplementary material for: DNA Damage Triggers Genetic Exchange in Helicobacter pylori
Source: PLoS Pathog. 2010 Jul 29;6(7):e1001026. doi: 10.1371/journal.ppat.1001026 (PMC2912397; doi:10.1371/journal.ppat.1001026)
Supplement: Table S1 — Similar genes are induced in cells treated with ciprofloxacin and in the ΔaddA mutant. All genes listed are significantly induced by SAM, using a 1% FDR for ciprofloxacin and a 5% FDR for the ΔaddA mutant. DNA damage regulon genes are highlighted in bold. Induced transcripts are listed in genome order for the strain G27 [46]. (0.22 MB DOC) [file ppat.1001026.s002.doc]

**Table S1**

Table S1: Similar genes are induced in cells treated with ciprofloxacin and in the ∆*addA* mutant.

| Induced, ciprofloxacin | Induced, ∆*addA* | Function |
| --- | --- | --- |
| **HPG27_15 *comB3*** | **HPG27_15 *comB3*** | competence apparatus |
| HPG27_16 *comB4* |  | competence apparatus |
| HPG27_30 *clpA* |  | protein degradation |
| **HPG27_36 *comB9*** | **HPG27_36 *comB9*** | competence apparatus |
| HPG27_42 *hypF* |  | regulatory |
| HPG27_57 |  | hypothetical protein |
| **HPG27_60** | **HPG27_60** | hypothetical protein |
| **HPG27_73** | **HPG27_73** | hypothetical protein |
|  | HPG27_76 *rps9* | ribosomal protein |
|  | HPG27_78 | hypothetical protein |
| HPG27_81 *rpoD* |  | transcriptional regulation |
| **HPG27_110** | **HPG27_110** | hypothetical protein |
| HPG27_111 *ppsA* |  | glycolysis/gluconeogenesis |
| HPG27_114 *rpl35* |  | ribosomal protein |
| HPG27_133 *ccoQ* |  | electron transport |
| HPG27_135 |  | hypothetical protein |
| HPG27_153 |  | hypothetical protein |
|  | HPG27_186 | hypothetical protein |
| HPG27_188 |  | hypothetical protein |
| HPG27_196 huNaDC-1 |  | transport |
| **HPG27_203** | **HPG27_203** | hypothetical protein |
| HPG27_222 |  | hypothetical protein |
| HPG27_233 |  | hypothetical protein |
| **HPG27_243 *clpB*** | **HPG27_243 *clpB*** | protein degradation |
| HPG27_247 |  | hypothetical protein |
| HPG27_256 |  | electron transport |
| HPG27_270 |  | hypothetical protein |
|  | HPG27_276 *rpl27* | ribosomal protein |
| HPG27_286 |  | hypothetical protein |
| HPG27_312 *ilvC* |  | amino acid biosynthesis, pyruvate family |
| **HPG27_314 *minE*** | **HPG27_314 *minE*** | cell division |
| **HPG27_320** | **HPG27_320** | lysozyme-like |
| HPG27_321 |  | hypothetical protein |
| HPG27_322 |  | hypothetical protein |
| HPG27_327 |  | hypothetical protein |
| **HPG27_340 *ftsK*** | **HPG27_340 *ftsK*** | cell division |
| HPG27_341 *addB* |  | recombination |
| HPG27_355 *copP* |  | transport and binding proteins |
| **HPG27_358** | **HPG27_358** | hypothetical protein |
| HPG27_365 |  | hypothetical protein |
| **HPG27_373** | **HPG27_373** | hypothetical protein |
| **HPG27_380 *infB*** | **HPG27_380 *infB*** | translation factor |
|  | HPG27_437 | hypothetical protein |
| HPG27_445 |  | hypothetical protein |
| HPG27_450 |  | transport and binding proteins |
| **HPG27_451 *rpl28*** | **HPG27_451 *rpl28*** | Ribosomal protein |
| HPG27_456 |  | hypothetical protein |
| HPG27_461 *gyrB* |  | DNA gyrase |
| HPG27_462 |  | hypothetical protein |
| HPG27_511 *rpl31* |  | Ribosomal protein |
| **HPG27_519 *acpP*** | **HPG27_519 *acpP*** | fatty acid and phospholipid metabolism |
| HPG27_519 *acpP* |  | fatty acid and phospholipid metabolism |
| HPG27_554 |  | hypothetical protein |
| HPG27_572 |  | hypothetical protein |
| HPG27_591 *mda66* |  | toxin production and resistance |
| HPG27_593 *hyaB* |  | electron transport |
| **HPG27_602** | **HPG27_602** | hypothetical protein |
| HPG27_617 |  | Cell envelope |
| **HPG27_636** | **HPG27_636** | hypothetical protein |
| HPG27_638 *nrdA* |  | 2'-deoxyribonucleotide metabolism |
| HPG27_639 |  | hypothetical protein |
| **HPG27_642 *fliP*** | **HPG27_642 *fliP*** | surface structures |
| HPG27_642 *fliP* |  | surface structures |
| HPG27_645 |  | hypothetical protein |
| **HPG27_669 *fic*** | **HPG27_669 *fic*** | cell division |
| HPG27_675 |  | hypothetical protein |
| **HPG27_675** | **HPG27_675** | hypothetical protein |
| HPG27_694 *ddl* |  | Biosynthesis of peptidoglycan |
| HPG27_732 *spoT* |  | stringent response |
| HPG27_733 |  | hypothetical protein |
| **HPG27_736 *acnB*** | **HPG27_736 *acnB*** | TCA cycle |
|  | HPG27_757 *moaD* | molybdopterin converting factor |
|  | HPG27_786 | hypothetical protein |
| **HPG27_795** | **HPG27_795** | hypothetical protein |
|  | HPG27_797 *ompP1* | outer membrane protein |
| HPG27_804 |  | hypothetical protein |
| HPG27_824 *flgE* |  | surface structures |
|  | HPG27_827 | hypothetical protein |
| HPG27_829 *katA* |  | catalase |
| **HPG27_832** | **HPG27_832** | hypothetical protein |
| **HPG27_841** | **HPG27_841** | hypothetical protein |
| HPG27_846 |  | hypothetical protein |
|  | HPG27_853 *hypC* | central intermediary metabolism |
| **HPG27_855** | **HPG27_855** | hypothetical protein |
| HPG27_861 HINDIIM |  | restriction enzyme |
| HPG27_865 |  | hypothetical protein |
| HPG27_873 *dmpI* |  | 4-oxalocrotonate tautomerase |
| HPG27_875 |  | hypothetical protein |
| **HPG27_880** | **HPG27_880** | hypothetical protein |
| **HPG27_887** | **HPG27_887** | hypothetical protein |
| HPG27_913 |  | hypothetical protein |
| HPG27_929 |  | hypothetical protein |
|  | HPG27_930 *xseA* | single strand exonuclease |
| **HPG27_934** | **HPG27_934** | hypothetical protein |
| HPG27_944 |  | hypothetical protein |
| HPG27_995 *pheT* |  | Amino acyl tRNA synthetases |
| **HPG27_1011** | **HPG27_1011** | hypothetical protein |
| HPG27_1012 |  | hypothetical protein |
| HPG27_1019 *ycf5* |  | electron transport |
| HPG27_1057 |  | hypothetical protein |
| **HPG27_1067** | **HPG27_1067** | hypothetical protein |
| HPG27_1084 *soj* |  | cell division |
| HPG27_1089 |  | hypothetical protein |
| HPG27_1096 *ffh* |  | protein and peptide secretion |
| HPG27_1098 |  | hypothetical protein |
| **HPG27_1107** | **HPG27_1107** | hypothetical protein |
|  | HPG27_1116 *glnH* | transport and binding proteins |
|  | HPG27_1145 *rpl7.12* | ribosomal protein |
| **HPG27_1146 *rpl10*** | **HPG27_1146 *rpl10*** | ribosomal protein |
| **HPG27_1151 *rpl33*** | **HPG27_1151 *rpl33*** | ribosomal protein |
| HPG27_1157 *cysE* |  | Amino acid biosynthesis, serine family |
| **HPG27_1161** | **HPG27_1161** | hypothetical protein |
| HPG27_1162 |  | hypothetical protein |
|  | HPG27_1182 | energy metabolism |
| HPG27_1186 |  | hypothetical protein |
| **HPG27_1189 *rps18*** | **HPG27_1189 *rps18*** | ribosomal protein |
|  | HPG27_1199 *bioC* | biotin biosynthesis |
|  | HPG27_1219 pflA | surface structures |
|  | HPG27_1243 *rpoA* | transcription |
| **HPG27_1247 *infA*** | **HPG27_1247 *infA*** | translation factor |
|  | HPG27_1248 *map* | translation factor |
|  | HPG27_1249 *secY* | protein and peptide secretion |
|  | HPG27_1250 *rpl15* | ribosomal protein |
|  | HPG27_1252 *rpl18* | ribosomal protein |
| HPG27_1259 *rps17* |  | ribosomal protein |
| HPG27_1260 *rpl29* |  | ribosomal protein |
|  | HPG27_1264 *rps19* | ribosomal protein |
|  | HPG27_1268 *rpl3* | ribosomal protein |
|  | HPG27_1269 *rps10* | ribosomal protein |
| HPG27_1277 *czcA* |  | transport and binding proteins |
|  | HPG27_1304 *nadA* | biosynthesis of pyridine nucleotides |
| **HPG27_1351** | **HPG27_1351** | hypothetical protein |
| **HPG27_1357** | **HPG27_1357** | hypothetical protein |
|  | HPG27_1370 | hypothetical protein |
| HPG27_1381 |  | electron transport |
| HPG27_1391 *ilvE* |  | amino acid biosynthesis, pyruvate family |
| HPG27_1405 |  | hypothetical protein |
|  | HPG27_1421 | hypothetical protein |
| HPG27_1425 |  | hypothetical protein |
| HPG27_1435 |  | hypothetical protein |
| HPG27_1436 *frpB* |  | transport and binding proteins |
| HPG27_1441 |  | hypothetical protein |
| HPG27_1452 |  | hypothetical protein |
| HPG27_1473 |  | hypothetical protein |
| **HPG27_1476 *petC*** | **HPG27_1476 *petC*** | electron transport |
| HPG27_1489 |  | hypothetical protein |
| HPG27_1496 *flgC* |  | surface structures |
| HPG27_1500 *tsaA* |  | detoxification |
|  | HPG27_1521 *gcp* | protein degradation |
| HPG27_1524 |  | hypothetical protein |
| HPG27_1525 |  | hypothetical protein |
| HPG27_1526 |  | hypothetical protein |
| HPG27_1564 |  | hypothetical protein |
| HPG27_1695 |  | hypothetical protein |
| HPG27_1696 |  | hypothetical protein |

All genes listed are significantly induced by SAM, using a 1% FDR for ciprofloxacin and a 5% FDR for the ∆*addA* mutant. Independent clones of the ∆*addA* mutant marked with different antibiotic resistance cassettes gave similar transcriptional profiles. DNA damage regulon genes are highlighted in bold. Induced transcripts are listed in genome order for the strain G27 [1].
