# Supplementary material for: DNA Damage Triggers Genetic Exchange in Helicobacter pylori
Source: PLoS Pathog. 2010 Jul 29;6(7):e1001026. doi: 10.1371/journal.ppat.1001026 (PMC2912397; doi:10.1371/journal.ppat.1001026)
Supplement: Table S2 — The ΔrecR mutant shows no significant transcriptional induction, although there are genes showing greater than 1.6-fold induction by microarray in the ΔrecR mutant relative to wild-type cells. No genes listed are significantly induced by SAM at 1% FDR and no FDR below 85% generates significant changes from wild-type cells. Independent clones of the ΔrecR mutant marked with different antibiotic resistance cassettes gave similar transcriptional profiles. (0.20 MB DOC) [file ppat.1001026.s003.doc]

**Table S2:** The ∆*recR* mutant shows no significant transcriptional induction, although there are genes showing greater than 1.6-fold induction by microarray in the ∆*recR* mutant relative to wild-type cells.

| G27 ID | Function | average fold change (n=3) | standard deviation |
| --- | --- | --- | --- |
| HpG27_564 | Hypothetical | 23.94 | 39.92 |
| HPG27_1055 | *omp24*, cell envelope | 3.35 | 3.92 |
| HPG27_0018 | Hypothetical | 3.29 | 3.78 |
| HPG27_657 | *gyrA,* DNA metabolism, DNA replication, recombination, and repair | 3.15 | 4.11 |
| HPG27_544 | *nth*, DNA metabolism, DNA replication, recombination, and repair | 2.43 | 1.81 |
| HPG27_591 | *mda66*, Cellular Processes, Toxin production and resistance | 2.42 | 2.38 |
| HPG27_509 | Cell envelope, Biosynthesis of murein sacculus and peptidoglycan | 2.19 | 1.86 |
| HPG27_1115 | Toxin production and resistance | 2.12 | 0.95 |
| HPG27_1276 | *glnQ*, Transport and binding proteins, Amino acids, peptides and amines | 2.09 | 1.23 |
| HPG27_1276 | *czcA*, Transport and binding proteins | 2.06 | 1.15 |
| HPG27_1255 | *rpS14*, Translation, Ribosomal proteins: synthesis and modification | 1.97 | 2.09 |
| HPG27_976 | Hypothetical | 1.96 | 1.34 |
| HPG27_1257 | rpl24, Translation, Ribosomal proteins: synthesis and modification | 1.92 | 1.78 |
| HPG27_1349 | Hypothetical | 1.91 | 0.93 |
| HPG27_84 | *hsdR,* Restriction/modification | 1.91 | 1.69 |
| HPG27_1393 | polA, DNA metabolism | 1.90 | 1.43 |
| HPG27_766 | Hypothetical | 1.90 | 1.12 |
| HPG27_1247 | *infA,* Translation, Translation factors | 1.90 | 1.95 |
| HPG27_1275 | Hypothetical | 1.89 | 0.89 |
| HPG27_1146 | *rpl10,* Translation, Ribosomal proteins | 1.86 | 1.81 |
| HPG27_894 | Hypothetical | 1.84 | 1.31 |
| HPG27_1425 | *omp32*, Cell envelope | 1.84 | 1.39 |
| HPG27_1264 | *rps19*, Translation, Ribosomal proteins | 1.79 | 1.70 |
| HPG27_1265 | *rpl2*, Translation, Ribosomal proteins | 1.79 | 1.80 |
| HPG27_1248 | Translation, Protein modification | 1.78 | 1.58 |
| HPG27_1350 | *kpsF,* Cell envelope, Biosynthesis of surface polysaccharides | 1.78 | 0.68 |
| HPG27_370 | *panB*, biosynthesis of cofactors, Pantothenate | 1.78 | 1.64 |
| HPG27_735 | Hypothetical | 1.73 | 1.39 |
| HPG27_11 | *dnaG*, DNA metabolism, DNA replication, recombination, and repair | 1.72 | 0.89 |
| HPG27_837 | Hypothetical | 1.72 | 0.88 |
| HPG27_1482 | *tagE*, Cell envelope, Biosynthesis of murein sacculus and peptidoglycan | 1.71 | 0.43 |
| HPG27_307 | *neuA*, Cell envelope, Biosynthesis of surface polysaccharides | 1.71 | 1.36 |
| HPG27_1256 | rpl5, Translation, Ribosomal proteins: synthesis and modification | 1.70 | 1.33 |
| HPG27_757 | *moaD*, biosynthesis of cofactors, prosthetic groups and carriers | 1.69 | 1.22 |
| HpG27_229 | Hypothetical | 1.68 | 0.55 |
| HPG27_1073 | Hypothetical | 1.67 | 1.02 |
| HPG27_1145 | rpl7/l12, Translation, Ribosomal proteins | 1.66 | 1.37 |
| HPG27_76 | *rps9*, Translation, Ribosomal proteins | 1.65 | 1.31 |
| HPG27_829 | *katA,* Detoxification | 1.64 | 1.58 |
| HPG27_1437 | *selA*, Translation, tRNA modification | 1.64 | 0.90 |
| HPG27_1249 | *secY,* Protein and peptide secretion | 1.63 | 1.31 |
| HPG27_1315 | Hypothetical | 1.62 | 0.91 |
| HPG27_726 | *mobA*, biosynthesis of cofactors, prosthetic groups and carriers | 1.62 | 0.60 |
| HPG27_1202 | Hypothetical | 1.61 | 1.10 |
| HPG27_615 | Transport and binding proteins | 1.61 | 1.25 |
| HPG27_315 | Hypothetical | 1.61 | 0.65 |

No genes listed are significantly induced by SAM at 1% FDR and no FDR below 85% generates significant changes from wild-type cells. Independent clones of the ∆*recR* mutant marked with different antibiotic resistance cassettes gave similar transcriptional profiles.
