# Supplementary material for: DNA Damage Triggers Genetic Exchange in Helicobacter pylori
Source: PLoS Pathog. 2010 Jul 29;6(7):e1001026. doi: 10.1371/journal.ppat.1001026 (PMC2912397; doi:10.1371/journal.ppat.1001026)
Supplement: Table S3 — Gene set analysis of gene ontology (GO) terms for ciprofloxacin treated cells. Terms listed have p<0.03, using the generation1 gene ontology (Materials and Methods). (0.05 MB DOC) [file ppat.1001026.s004.doc]

**Table S3**: Gene set analysis of gene ontology (GO) terms for ciprofloxacin treated cells.

| GO term | GO number | z-statistic |
| --- | --- | --- |
| ribosomal subunit | GO:0033279 | 7.1 |
| catalytic activity | GO:0003824 | 12.2 |
| genetic transfer | GO:0009292 | 7.1 |
| aminoacyl-tRNA ligase activity | GO:0004812 | 7.7 |
| biological_process | GO:0008150 | 7.6 |
| DNA binding | GO:0003677 | 6.2 |
| guanyl ribonucleotide binding | GO:0032561 | 9.7 |
| nucleoside-triphosphatase activity | GO:0017111 | 10.5 |
| primary metabolic process | GO:0044238 | 6.0 |
| metal ion binding | GO:0046872 | 14.0 |
| external encapsulating structure organization | GO:0045229 | 5.6 |
| binding | GO:0005488 | 11.3 |
| cell projection | GO:0042995 | 6.8 |
| hydrogen ion transmembrane transporter activity | GO:0015078 | 5.0 |
| intracellular part | GO:0044424 | 19.6 |

Terms listed have p<0.03, using the generation1 gene ontology (Materials and Methods).
