# Supplementary material for: DNA Damage Triggers Genetic Exchange in Helicobacter pylori
Source: PLoS Pathog. 2010 Jul 29;6(7):e1001026. doi: 10.1371/journal.ppat.1001026 (PMC2912397; doi:10.1371/journal.ppat.1001026)
Supplement: Table S4 — Genes significantly induced in wild-type cells treated with gentamicin (SAM, 1% FDR). DNA damage regulon genes are highlighted in bold. Induced transcripts are listed in genome order for the strain G27 [46]. (0.11 MB DOC) [file ppat.1001026.s005.doc]

**Table S4:** Genes significantly induced in wild-type cells treated with gentamicin (SAM, 1% FDR).

| **Induced** | **Function** |
| --- | --- |
| HPG27_10 dnaG | DNA primase |
| HPG27_76 | phosphoglycerate dehydrogenas |
| HPG27_81 | hypothetical |
| HPG27_98 | cystathionine gamma-synthase |
| HPG27_99 cysK | cysteine synthetase |
| HPG27_101 | hypothetical |
| HPG27_102 sdaA | L-serine deaminase |
| HPG27_103 sdaC | serine transporte |
| **HPG27_110** | hypothetical |
| HPG27_111 | phosphoenol pyruvate synthase |
| HPG27_131 | hypothetical |
| HPG27_133 moeA | molybdopterin biosynthesis protein |
| HPG27_196 omp8 | outer membrane protein |
| **HPG27_203 clpB** | ATP-dependent protease binding subunit |
| HPG27_215 | hypothetical |
| HPG27_219 | hypothetical |
| HPG27_276 fliG | flagellar motor switch protein |
| HPG27_311 sodB | superoxide dismutase |
| HPG27_327 | hypothetical |
| HPG27_1021 | ferrochelatase |
| HPG27_1015 | zinc-metalloprotease |
| HPG27_994 | phenylalanyl-tRNA synthetase, alpha subunit |
| HPG27_990 | catalase-like |
| HPG27_440 | hypothetical membrane protein |
| HPG27_445 | hypothetical |
| HPG27_457 oorB | ferroredoxin |
| **HPG27_519** | hypothetical |
| HPG27_523 | hypothetical |
| HPG27_594 mreB | rod shape-determining protein |
| HPG27_599 omp13 | outer membrane protein |
| HPG27_638 | ribonucleoside-diphosphatereductase 1 alpha subunit |
| HPG27_644 | iron (II) transport protein |
| HPG27_744 | glyceraldehyde-3-phosphate dehydrogenase |
| HPG27_804 | hypothetical |
| HPG27_758 | hypothetical |
| HPG27_783 | hypothetical |
| HPG27_869 | hypothetical |
| HPG27_411 | hypothetical |
| HPG27_410 hisS | histidyl-tRNA synthetase |
| HPG27_409 rfaF | ADP-heptose-lps heptosyltransferase II |
| HPG27_351 | hypothetical |
| HPG27_1040 | hypothetical |
| HPG27_1044 | hypothetical |
| HPG27_1045 rpoA | DNA-directed RNA polymerase |
| HPG27_1117 mreB | rod shape-determining protein |
| HPG27_1146 hsdM | type I restriction enzyme M protein |
| HPG27_1189 | hypothetical |
| HPG27_1190 | hypothetical |
| HPG27_1191 | hypothetical |
| HPG27_1243 | DNA-directed RNA polymerase, alpha subunit |
| **HPG27_1247** | hypothetical |
| HPG27_1248 ECO57IR | type IIS restriction enzyme R and M protein |
| HPG27_1249 | hypothetical |
| HPG27_1250 | hypothetical |
| HPG27_1251 | type III restriction enzyme |
| HPG27_1252 | hypothetical |
| HPG27_1256 dnaA | chromosomal replication initiator protein |
| HPG27_1257 glmS | glucosamine fructose-6-phosphate aminotransferase |
| HPG27_1260 tnpA | ransposase |
| HPG27_1261 | hypothetical |
| HPG27_1262 | hypothetical |
| HPG27_1263 petC | cytochrome c1 subunit |
| HPG27_1264 petB | cytochrome b subunit |
| HPG27_1267 tagE | toxR-activated gene |
| HPG27_1269 | hypothetical |
| HPG27_1282 | hypothetical |
| HPG27_1321 | hypothetical |
| HPG27_1459 | hypothetical |
| HPG27_1346 | hypothetical |
| HPG27_1367 | non-functional restriction enzyme |
| HPG27_1399 oppD | oligopeptide ABC transporter |
| HPG27_1402 xseA | exonuclease |
| HPG27_1415 | hypothetical |
| HPG27_1420 | hypothetical |
| HPG27_1448 | hypothetical |
| HPG27_1476 rnhA | RIBONUCLEASE HI |
| HPG27_1477 | hypothetical |
| HPG27_1492 fliL | FLAGELLAR BIOSYNTHESIS PROTEIN |
|  |  |

DNA damage regulon genes are highlighted in bold.
