# Supplementary material for: DNA Damage Triggers Genetic Exchange in Helicobacter pylori
Source: PLoS Pathog. 2010 Jul 29;6(7):e1001026. doi: 10.1371/journal.ppat.1001026 (PMC2912397; doi:10.1371/journal.ppat.1001026)
Supplement: Table S5 — Genes significantly induced in comB4IE cells (SAM, 1% FDR). DNA damage regulon genes are highlighted in bold. Induced transcripts are listed in genome order for the strain G27 [46]. (0.21 MB DOC) [file ppat.1001026.s006.doc]

**Table S5:** Genes significantly induced in *comB4^IE^* cells (SAM, 1% FDR).

| Induced, *comB4IE* | Function |
| --- | --- |
| HPG27_11 *dnaG* | replication |
| **HPG27_15 *comB3*** | competence apparatus |
| HPG27_17 | hypothetical protein |
| HPG27_22 *omp2* | cell envelope |
| **HPG27_36 *comB9*** | competence apparatus |
| HPG27_41 | central intermediary metabolism |
| HPG27_56 | hypothetical protein |
| **HPG27_73** | hypothetical protein |
| HPG27_81 *rpoD* | transcriptional regulator |
| HPG27_87 | hypothetical protein |
| **HPG27_110** | hypothetical protein |
| HPG27_114 *rpl35* | ribosomal protein |
| HPG27_133 *ccoQ* | electron transport |
| HPG27_135 | hypothetical protein |
| HPG27_157 *moeA* | molybdopterin |
| HPG27_196 *rpl32* | ribosomal protein |
| HPG27_186 | hypothetical protein |
| HPG27_188 | hypothetical protein |
| HPG27_203 | hypothetical protein |
| HPG27_210 | hypothetical protein |
| HPG27_222 | hypothetical protein |
| HPG27_224 *atoS* | regulatory |
| HPG27_229 | hypothetical protein |
| **HPG27_233 *omp26*** | cell envelope |
| HPG27_239 *mod* | methyltransferase |
| **HPG27_243 *clpB*** | protein degradation |
| HPG27_245 | hypothetical protein |
| HPG27_247 | hypothetical protein |
| HPG27_256 | electron transport |
| HPG27_260 *tgt* | tRNA modification |
| HPG27_261 | hypothetical protein |
| HPG27_262 *aroB* | amino acid biosynthesis, aromatic family |
| HPG27_286 | hypothetical protein |
| HPG27_309 *flaG* | surface structure |
| HPG27_311 n*adE* | pyridine nucleotide biosynthesis |
| HPG27_315 *dprA* | natural transformation |
| **HPG27_320 *lys*** | lysozyme-like |
| HPG27_322 | hypothetical protein |
| HPG27_323 | hypothetical protein |
| HPG27_328 | surface structure |
| **HPG27_340 *ftsK*** | cell division |
| HPG27_341 *addB* | recombination and repair |
| HPG27_344 *hlyA* | hypothetical protein |
| HPG27_345 | hypothetical protein |
| HPG27_355 *fliR* | surface structure |
| **HPG27_358** | hypothetical protein |
| HPG27_364 | hypothetical protein |
| HPG27_366 | tRNA modification |
| **HPG27_373** | hypothetical protein |
| HPG27_374 | hypothetical protein |
| HPG27_382 | hypothetical protein |
| HPG27_387 *flhA* | surface structure |
| HPG27_411 *rocE* | transport and binding protein |
| HPG27_417 *pyrD* | pyrimidine ribonucleotide biosynthesis |
| HPG27_418 *ppk* | Central intermediary metabolism |
| HPG27_421 *hsdR* |  |
| HPG27_422 *hsdR* | restriction enzyme |
| HPG27_437 | hypothetical protein |
| HPG27_447 | hypothetical protein |
| HPG27_461 *gyrB* | DNA metabolism |
| HPG27_462 | hypothetical protein |
| HPG27_478 | hypothetical protein |
| HPG27_483 *cag5* | cag apparatus |
| HPG27_496 *cag17* | cag apparatus |
| HPG27_498 *cag19* | cag apparatus |
| **HPG27_519 *acpP*** | fatty acid biosynthesis |
| HPG27_523 | hypothetical protein |
| HPG27_542 | hypothetical protein |
| HPG27_547 *pabc* | folic acid biosynthesis |
| HPG27_554 | hypothetical protein |
| HPG27_564 | hypothetical protein |
| HPG27_606 | hypothetical protein |
| HPG27_625 *aroC* | amino acid biosynthesis, aromatic family |
| HPG27_630 | hypothetical protein |
| HPG27_641 *glmU* | sugar-nucleotide biosynthesis |
| HPG27_642 *fliP* | surface structure |
| HPG27_645 | hypothetical protein |
| HPG27_652 | Central intermediary metabolism |
| **HPG27_669 *fic*** | cell division |
| HPG27_671 | transport and binding protein |
| **HPG27_675** | hypothetical protein |
| HPG27_682 | regulatory |
| HPG27_703 | hypothetical protein |
| HPG27_704 | hypothetical protein |
| HPG27_712 *moeB* | molybdopterin |
| HPG27_716 | hypothetical protein |
| HPG27_733 | hypothetical protein |
| HPG27_742 *secA* | protein and peptide secretion |
| HPG27_757 *moaD* | molybdopterin |
| HPG27_776 | methyltransferase |
| HPG27_782 | hypothetical protein |
| HPG27_794 *hup* | translation |
| **HPG27_795** | hypothetical protein |
| HPG27_795 | hypothetical protein |
| HPG27_804 | hypothetical protein |
| HPG27_821 *lpxB* | biosynthesis of surface polysaccharides |
| HPG27_827 | hypothetical protein |
| HPG27_834 | hypothetical protein |
| HPG27_837 | hypothetical protein |
| HPG27_840 *vacA* | toxin production |
| HPG27_846 | hypothetical protein |
| HPG27_848 | hypothetical protein |
| HPG27_853 *hypC* | Central intermediary metabolism |
| HPG27_858 | surface structure |
| HPG27_859 | surface structure |
| HPG27_862 | replication |
| HPG27_863 | cell envelope |
| HPG27_864 | cell envelope |
| HPG27_873 | hypothetical protein |
| HPG27_882 | hypothetical protein |
| **HPG27_887** | hypothetical protein |
| HPG27_912 | hypothetical protein |
| HPG27_919 *glyS* | tRNA synthetase |
| **HPG27_935** | hypothetical protein |
| HPG27_947 | hypothetical protein |
| HPG27_949 | hypothetical protein |
| HPG27_990 | biotin biosynthesis |
| HPG27_991 | hypothetical protein |
| HPG27_992 | hypothetical protein |
| **HPG27_1011** | hypothetical protein |
| HPG27_1012 | hypothetical protein |
| HPG27_1013 | hypothetical protein |
| HPG27_1019 *ycf5* | electron transport |
| HPG27_1023 | hypothetical protein |
| HPG27_1055 *omp24* | cell envelope |
| HPG27_1056 *uvrB* | recombination and repair |
| HPG27_1064 *flgK* | surface structure |
| HPG27_1088 | hypothetical protein |
| HPG27_1097 *valS* | tRNA synthetase |
| **HPG27_1151 *rpl33*** | translation factor |
| HPG27_1167 | hypothetical protein |
| HPG27_1176 *folP* | folic acid biosynthesis |
| HPG27_1186 | hypothetical protein |
| **HPG27_1189 *rps18*** | ribosomal protein |
| HPG27_1211 *nqo3* | energy metabolism, aerobic |
| HPG27_1228 *trpE* | amino acid biosynthesis, aromatic family |
| **HPG27_1247 infA** | translation factor |
| HPG27_1260 *rpl29* | ribosomal protein |
| HPG27_1270 | hypothetical protein |
| HPG27_1278 | hypothetical protein |
| HPG27_1306 | hypothetical protein |
| HPG27_1313 | regulatory |
| HPG27_1316 | restriction enzyme |
| HPG27_1324 *lon* | degradation of proteins |
| HPG27_1473 | hypothetical protein |
| HPG27_1468 | hypothetical protein |
| HPG27_1328 | restriction enzyme |
| **HPG27_1351** | hypothetical protein |
| **HPG27_1357** | hypothetical protein |
| HPG27_1401 *rep* | replication |
| HPG27_1405 | hypothetical protein |
| HPG27_1416 | hypothetical protein |
| HPG27_1435 | hypothetical protein |
| HPG27_1436 *frpB* | transport and binding protein |
| HPG27_1445 *recG* | recombination and repair |
| HPG27_1448 *exoA* | recombination and repair |
| HPG27_1452 | hypothetical protein |
| HPG27_1489 | hypothetical protein |
| HPG27_1502 *pbp2* | cell envelope |
| HPG27_1508 *rlpA* | cell envelope |
| HPG27_1524 | hypothetical protein |
| HPG27_1525 | hypothetical protein |
| HPG27_1526 | hypothetical protein |
| HPG27_1564 | hypothetical protein |
| HPG27_1688 | hypothetical protein |
| HPG27_1695 | hypothetical protein |
| HPG27_1696 | hypothetical protein |

DNA damage regulon genes are highlighted in bold. Induced transcripts are listed in genome order for the strain G27 [1].
