# Supplementary material for: DNA Damage Triggers Genetic Exchange in Helicobacter pylori
Source: PLoS Pathog. 2010 Jul 29;6(7):e1001026. doi: 10.1371/journal.ppat.1001026 (PMC2912397; doi:10.1371/journal.ppat.1001026)
Supplement: Table S6 — H. pylori strains used in these studies H. pylori strains used in these studies (0.03 MB DOC) [file ppat.1001026.s007.doc]

Table S6: *H. pylori* strains used in these studies

| Genotype | alias | Reference or source |
| --- | --- | --- |
| *∆addA::cat* | HpG27_1491 | [2] |
| *∆addA::aph3* | HpG27_1491 | this work |
| *∆comB10::cat* | HpG27_37 | this work |
| *∆addA::aph3 ∆comB10::cat* |  | this work |
| *∆recA::cat* | HpG27_140 | [2] |
| *rdxA::comB4* | *comB4^IE^,* HPG27_16 | this work |
| *rdxA::comB4 comB10::cat* |  | this work |
| *rdxA::comB4 recA::cat* |  | this work |
| *∆lys:cat* | HpG27_320 | this work |
| *∆lys:cat rdxA::lys* |  | this work |
