# Supplementary material for: DNA Damage Triggers Genetic Exchange in Helicobacter pylori
Source: PLoS Pathog. 2010 Jul 29;6(7):e1001026. doi: 10.1371/journal.ppat.1001026 (PMC2912397; doi:10.1371/journal.ppat.1001026)
Supplement: Table S7 — Oligonucleotides used in this study. Gene specific sequences are in upper case and sequences added for cloning in lower case. *Denotes oligos used for qPCR. All others were used to generate deletions # denotes oligos also used to generate complementation plasmids. (0.05 MB DOC) [file ppat.1001026.s008.doc]

Table S7: Oligonucleotides used in this study.

| Name | Sequence |
| --- | --- |
| 1184* *comB9* | GCGCTTTAAAAGCCGATG |
| 1185* *comB9* | GGTCGCCATCGCATAA |
| 1186* *comB8* | GATGAGCTTTATCGCGC |
| 1187* *comB8* | AGCGCGATTTATCGTCAA |
| 1209* *recA* | GCATGATGGGTTATGGG |
| 1210* *recA* | TCCCCAAACATGATGTCA |
| 1007 *comB10#* | gctctagaGGATTGTGGGCGATTA |
| 1008 *comB10#* | acgcgtcgacGAGCGTTTCATCTACTAA |
| 1009 *comB10* | atccacttttcaatctatatcGCTGTTCTTGTAAGGC |
| 1010 *comB10* | cccagtttgtcgcactgataaGAGACAAGAGCAAGATCG |
| 1090 *comB4#* | gcgcgctctagaCGCTCTTTAGCGTGCTG |
| 1091 *comB4#* | gcgcgcgtcgacCTAGCTCTTCAAAAGTTTC |
| 1228 *lys#* | gctctagaCAGAGCCAAGAAACCC |
| 1229 *lys#* | acgcgtcgacTCCTTTACAAAAGTTAAGAC |
| 1230 *lys* | atccacttttcaatctatatcATAGCCATAGCCAATCGT |
| 1231 *lys* | cccagtttgtcgcactgataaGCGAGAGATTTTGAAAGAA |

Gene specific sequences are in uppercase and sequences added for cloning in lower case. *Denotes oligos used for qPCR. All others were used to generate deletions ^#^ denotes oligos also used to generate complementation plasmids.
